# Supplementary material for: In-Flight Emergency: A Simulation Case for Emergency Medicine Residents
Source: MedEdPORTAL. 2020 Aug 20;16:10949. doi: 10.15766/mep_2374-8265.10949 (PMC7449573; doi:10.15766/mep_2374-8265.10949)
Supplement: Supplementary file 1 — Simulation Case.docxSimulation Images.docxMedical Kit Supply List.docxCritical Actions Checklist.docxResident Evaluation.docxLearning Points.docx [file mep_2374-8265.10949-s001.zip › A. Simulation Case.docx]

| **Appendix A: MedEdPORTAL Simulation Case Template**  **SIMULATION CASE TITLE: In-Flight Emergency: A Simulation Case for Emergency Medicine Residents**  **AUTHORS:** Charles Lei, MD, Claire Hailey, MD  **LEARNER AUDIENCE:** Emergency Medicine Residents | |
| --- | --- |
| **PATIENT AGE: 55 years old**  **CHIEF COMPLAINT: Shortness of breath**  **PHYSICAL SETTING: Airplane** | |
|  | |
| **Brief Narrative Description of Case** | The patient is a 55-year-old male with a history of COPD who develops shortness of breath and chest pain on a commercial flight. Learners will initiate care for the patient. They will delegate roles and tasks to team members as well as to the flight attendants. They will utilize resources including the in-flight emergency medical kit and AED. They should recognize the patient’s respiratory distress and formulate a differential diagnosis. The patient has developed a tension pneumothorax and will suffer a PEA arrest. Learners should follow ACLS protocol. They should consider and treat tension pneumothorax with needle thoracostomy. After needle decompression, a flight attendant will have a near syncopal episode. The learners should evaluate and treat the flight attendant. They should also communicate with the pilot and ground-based medical staff regarding the need to divert the plane to the nearest airport. |
| **Primary Learning Objectives** | By the end of this activity, learners will be able to:  1. Identify the resources available during an in-flight emergency  2. Describe the role of a medical professional responding to an in-flight emergency  3. Recognize the presentation of tension pneumothorax and describe its management  4. Demonstrate the appropriate management of pulseless electrical activity cardiac arrest  5. Demonstrate the appropriate initial evaluation and treatment of a patient with a near syncopal episode |
| **Critical Actions** | - Introduce team to flight attendants and passenger   - State name and medical qualifications   - Ask passenger for permission to treat - Establish team roles   - Identify team leader   - Delegate tasks to team members - Identify available resources   - Request in-flight emergency medical kit   - Perform inventory of medical kit   - Request AED   - Inquire about on-board oxygen supply   - Enlist flight attendants and/or other passengers - Manually obtain passenger’s vital signs   - Measure heart rate   - Obtain manual blood pressure   - Measure respiratory rate - Obtain vascular access - Recognize and treat passenger’s respiratory distress   - Perform physical exam   - Administer supplemental O2   - Administer bronchodilators - Perform appropriate resuscitative measures on passenger in PEA arrest   - Perform chest compressions   - Utilize AED   - Administer IV epinephrine   - Consider and treat tension pneumothorax (with needle thoracostomy) - Optimize positioning of passenger for resuscitation   - Move to aisle seat for management of respiratory distress   - Move to aisle or galley for management of PEA arrest - Assess and treat flight attendant with near-syncopal episode   - Manually obtain vital signs   - Measure blood glucose or administer dextrose   - Place in recumbent position   - Administer IV or oral fluids - Communicate and coordinate with pilot and ground-based medical service   - Provide update on status of passenger and flight attendant   - Advise diversion of airplane to nearest airport |
| **Learner Preparation** | Learners should be oriented to the simulation space and simulation personnel prior to the start of the case. |

| Initial Presentation | | | |
| --- | --- | --- | --- |
| **Initial Vital Signs** | HR 120, BP 90/60, RR 30 | | |
| **Overall Appearance** | The passenger is seated in the window seat in the second row of the cabin. He is in obvious respiratory distress, speaking in 3 to 4-word sentences, and coughing. | | |
| **Actors and Roles in the Room at Case Start** | Flight Attendant #1 (actor) announces: “Is there a doctor on board?”  Flight Attendant #2 (actor) is available for assistance. | | |
| **HPI** | The patient reports that he had sudden onset of shortness of breath and left-sided chest pain about 30 minutes after take-off. The chest pain is sharp, pleuritic, and does not radiate. Further history is limited by the patient’s dyspnea. | | |
| **Past Medical/Surgical History** | **Medications** | **Allergies** | **Family History** |
| COPD  Hypertension  Diabetes | Albuterol inhaler as needed  Amlodipine 5mg daily  Hydrochlorothiazide 25mg daily | None | None |
| **Physical Examination** | | | |
| **General** | Sitting in window seat, in respiratory distress | | |
| **HEENT** | PERRL, moist mucous membranes, oropharynx clear | | |
| **Neck** | Supple, no stridor | | |
| **Lungs** | Tachypneic, bilateral wheezing, decreased breath sounds on the left side | | |
| **Cardiovascular** | Tachycardic, regular rhythm, no murmurs | | |
| **Abdomen** | Soft, nontender, nondistended | | |
| **Neurological** | Alert and oriented x3, cranial nerves II-XII intact, moving all extremities | | |
| **Skin** | No rash or urticaria | | |
| **Psychiatric** | Anxious appearing | | |

| Instructor Notes - Changes and CASE Branch Points | | |
| --- | --- | --- |
| **Intervention / Time Point** | **Change in Case** | **Additional Information** |
| Time = 0 minutes | - Passenger vital signs: HR 120, BP 90/60, RR 30  - Passenger is short of breath, speaking in 3 to 4-word sentences, and coughing | - Flight Attendant #1 will start case by announcing: “Is there a doctor on board?” |
| Learner actions:  - Introduce team to flight attendants and passenger  - Establish team roles  - Manually obtain passenger’s vital signs  - Perform physical exam | - Passenger reports shortness of breath and chest pain  - Passenger has bilateral wheezing and decreased breath sounds on the left side |  |
| Time = 2 minutes | - Passenger vital signs: HR 140, BP 70/40, RR 40  - Passenger is progressively more short of breath and speaking in 1 to 2-word sentences | - Flight attendants will alert learners: “The passenger appears to be having more difficulty breathing!” |
| Learner actions:  - Identify available resources, including medical kit and AED  - Obtain vascular access  - Administer supplemental O2  - Administer bronchodilators  - Optimize positioning of passenger | - Passenger has no improvement with supplemental O2 or bronchodilators | - Flight Attendant #1 will provide medical kit and AED when requested  - Flight attendants can assist with moving passenger |
| Time = 5 minutes | - Passenger vital signs: HR 0, BP unmeasurable, RR 0  - Patient becomes unresponsive, pulseless, and apneic | - Flight attendants will alert learners: “The passenger is no longer responding to me!” |
| Learner actions:  - Optimize positioning of passenger  - Perform chest compressions  - Utilize AED  - Administer IV epinephrine  - Perform bag mask ventilation | - AED states: “No shock advised” | - Flight attendants can assist with moving passenger  - Flight attendants can assist with performing chest compressions |
| Time = 8 minutes | - Passenger remains in PEA arrest |  |
| Learner actions:  - Continue resuscitative measures on passenger  - Consider and treat tension pneumothorax with needle thoracostomy | - After needle decompression, passenger has ROSC: HR 120, BP 100/70, RR 20  - Immediately after witnessing needle thoracostomy, Flight Attendant #2 develops lightheadedness, palpitations, shortness of breath, and nausea  - Flight Attendant #2 stumbles and falls to floor | - Flight attendants will alert learners: “The passenger’s chest appears to be moving asymmetrically with bag mask ventilation.”  - Exact timing of needle decompression will vary depending on when learners recognize tension pneumothorax and may occur before Time = 8 minutes  - If learners do not perform needle thoracostomy, passenger will remain in PEA arrest until end of scenario  - If learners do not perform needle thoracostomy, Flight Attendant #2 will have near syncopal episode at Time = 8 minutes |
| Learner actions:  - Assess Flight Attendant #2  - Measure blood glucose and/or empirically administer dextrose  - Place in recumbent position  - Administer IV or oral fluids to Flight Attendant #2 | - Flight Attendant #2 vital signs: HR 120, BR 95/60, RR 25 | - Blood glucose is 95 mg/dL |
| Time = 12 minutes | - Passenger vital signs: HR 110, BP 150/80, RR 16  - Flight Attendant #2 vital signs: HR 85, BP 125/70, RR 12  - Flight Attendant #2 reports feeling better after receiving treatment | - If learners do not treat Flight Attendant #2, he/she will remain symptomatic until end of scenario |
| Learner actions:  - Communicate and coordinate with pilot and ground-based medical service | - When provided with an adequate explanation, ground-based physician provides approval for pilot to divert plane to nearest airport  - Pilot informs learners that he/she will initiate a rapid landing of airplane and an EMS crew will meet airplane on runway to transport passenger to nearest hospital | - Different learner groups may communicate with pilot and ground-based medical service at different time points in the case  - When initially asked to divert plane, pilot will ask learners to speak with ground-based medical staff  - Ground-based physician will request an explanation and provide approval for diversion only after passenger has suffered PEA arrest  - If learners have not communicated with pilot by Time = 12 minutes, pilot will request an update |

**Ideal Scenario Flow**

Flight Attendant #1 makes an announcement requesting medical assistance. Learners arrive to find a passenger in respiratory distress. They introduce themselves, state their medical qualifications, and obtain permission from the passenger to treat him. The passenger is able to state that he has shortness of breathing and chest pain, as well as a history of COPD. Learners immediately request the in-flight emergency medical kit and AED from the flight attendants. Learners obtain vital signs manually and find the passenger to be tachycardic, hypotensive, and tachypneic. They recognize that the patient has bilateral wheezing and diminished breath sounds on the left side. They establish IV access. They provide the passenger with supplemental oxygen and bronchodilators. The passenger’s respiratory distress does not improve. The passenger’s condition progressively worsens, until he becomes unresponsive, pulseless, and apneic. Learners immediately initiate CPR. They utilize the AED, which states “no shock advised.” They administer IV epinephrine and perform bag mask ventilation. Learners consider tension pneumothorax and perform needle thoracostomy, after which the passenger has ROSC. A flight attendant has a near syncopal episode after witnessing CPR and needle decompression. Learners administer fluids and empiric dextrose to the flight attendant and place him/her in a recumbent position. Learners discuss the passenger with the pilot and ground-based medical service, recommending diversion to the nearest airport for the patient to be transported to the nearest hospital. The case concludes with the pilot initiating a rapid landing of the airplane.

**Anticipated Management Mistakes**

1. Difficulty providing updated vital signs: We found when conducting this case that learners wanted to perform frequent manual reassessments of the passenger’s vital signs. To keep the case progressing forward, we experimented with having a flight attendant periodically announce the passenger’s vital signs, but felt that this method decreased the realism of this individual’s role. We found it helpful to display the vital signs using a mobile application (SimMon, Castle Anderson ApS) on a tablet device. The tablet served as a vital sign monitor that could be remotely controlled by a smartphone via Bluetooth. The vital signs were adjusted throughout the case when learners re-evaluated the passenger. We found that this method allowed us to efficiently provide learners with vital sign updates without compromising the realism of the case.
2. Failure to recognize and treat tension pneumothorax: Learners were given several clues that the passenger was suffering from a tension pneumothorax, including a history of COPD, a report of sudden-onset shortness of breath and left-sided chest pain, and an exam finding of diminished left-sided breath sounds. However, some learners did not consider that the passenger’s PEA arrest could be secondary to a tension pneumothorax. We found it helpful to have the flight attendants inform learners that the passenger’s chest wall was moving asymmetrically with bag mask ventilation. If learners still did not recognize tension pneumothorax, the passenger would remain in PEA arrest for the remainder of the case. We reviewed this topic with all learners during the debriefing session.
3. Uncertainty about general approach to responding to an in-flight emergency: Some learners were unfamiliar with the supplies available in an in-flight emergency medical kit and additional resources available during flight. In addition, many learners were uncertain of their role when responding to a call for medical assistance on an airplane. During the debriefing session we discussed how to approach an in-flight emergency and provided the learners with debriefing materials emphasizing this information.
