# Supplementary material for: In-Flight Emergency: A Simulation Case for Emergency Medicine Residents
Source: MedEdPORTAL. 2020 Aug 20;16:10949. doi: 10.15766/mep_2374-8265.10949 (PMC7449573; doi:10.15766/mep_2374-8265.10949)
Supplement: Supplementary file 1 — Simulation Case.docxSimulation Images.docxMedical Kit Supply List.docxCritical Actions Checklist.docxResident Evaluation.docxLearning Points.docx [file mep_2374-8265.10949-s001.zip › C. Medical Kit Supply List.docx]

In-Flight Emergency Medical Kit:

- Stethoscope

- Sphygmomanometer

- Gloves

- Self-inflating manual resuscitation device with masks (3 sizes: pediatric, small adult, large adult)

- Oropharyngeal airways (3 sizes: pediatric, small adult, large adult)

- CPR masks (3 sizes: pediatric, small adult, large adult)

- Tape scissors

- Needles (18-gauge, 20-gauge, 22-gauge; 2 each)

- Syringes (5cc, 10cc; 2 each)

- IV tubing with 2 Y-connectors

- IV starter kit with gauze, tourniquet, adhesive tape

- Antiseptic wipes

- Sharps disposal box

- Acetaminophen 325mg tablets

- Aspirin 325mg tablets

- Atropine 0.5mg, 5cc ampule

- Inhaled bronchodilator (metered dose inhaler)

- Dextrose 50%/50cc ampule

- Diphenhydramine 25mg tablets

- Diphenhydramine 50mmg injectable

- Epinephrine 1:1000 injectable

- Epinephrine 1:10000 injectable

- Lidocaine 20mg/ml, 5cc ampule

- Nitroglycerin 0.4mg tablets

- Normal saline 500cc

Also on board:

AED

Supplemental O2 supply
