# Supplementary material for: In-Flight Emergency: A Simulation Case for Emergency Medicine Residents
Source: MedEdPORTAL. 2020 Aug 20;16:10949. doi: 10.15766/mep_2374-8265.10949 (PMC7449573; doi:10.15766/mep_2374-8265.10949)
Supplement: Supplementary file 1 — Simulation Case.docxSimulation Images.docxMedical Kit Supply List.docxCritical Actions Checklist.docxResident Evaluation.docxLearning Points.docx [file mep_2374-8265.10949-s001.zip › D. Critical Actions Checklist.docx]

**CRITICAL ACTIONS**

- Introduce team to flight attendants and passenger
  - State name and medical qualifications
  - Ask passenger for permission to treat
- Establish team roles
  - Identify team leader
  - Delegate tasks to team members
- Identify available resources
  - Request in-flight emergency medical kit
  - Perform inventory of medical kit
  - Request AED
  - Inquire about on-board oxygen supply
  - Enlist flight attendants and/or other passengers
- Manually obtain passenger’s vital signs
  - Measure heart rate
  - Obtain manual blood pressure
  - Measure respiratory rate
- Obtain vascular access
- Recognize and treat passenger’s respiratory distress
  - Perform physical exam
  - Administer supplemental O2
  - Administer bronchodilators
- Perform appropriate resuscitative measures on passenger in PEA arrest
  - Perform chest compressions
  - Utilize AED
  - Administer IV epinephrine
  - Perform bag mask ventilation
  - Consider and treat tension pneumothorax (with needle thoracostomy)
- Optimize positioning of passenger for resuscitation
  - Move to aisle seat for management of respiratory distress
  - Move to aisle or galley for management of PEA arrest
- Assess and treat flight attendant with near-syncopal episode
  - Manually obtain vital signs
  - Measure blood glucose or administer dextrose
  - Place in recumbent position
  - Administer IV or oral fluids
- Communicate and coordinate with pilot and ground-based medical service
  - Provide update on status of passenger and flight attendant
  - Advise diversion of airplane to nearest airport
