# Supplementary material for: In-Flight Emergency: A Simulation Case for Emergency Medicine Residents
Source: MedEdPORTAL. 2020 Aug 20;16:10949. doi: 10.15766/mep_2374-8265.10949 (PMC7449573; doi:10.15766/mep_2374-8265.10949)
Supplement: Supplementary file 1 — Simulation Case.docxSimulation Images.docxMedical Kit Supply List.docxCritical Actions Checklist.docxResident Evaluation.docxLearning Points.docx [file mep_2374-8265.10949-s001.zip › E. Resident Evaluation.docx]

**Resident Simulation Course Evaluation**

**Date:**

**Faculty Instructor:**

**Simulation Topic: In-Flight Emergency**

**Level of Training:** PGY 1 _______ PGY 2 _______ PGY 3 _______

**1. Overall this was a valuable educational experience.**

(Strongly disagree) 1 2 3 4 5 (Strongly agree)

**2. The knowledge and skills gained from this course will change my practice.**

(Strongly disagree) 1 2 3 4 5 (Strongly agree)

**3. I feel prepared to respond to an in-flight emergency.**

(Strongly disagree) 1 2 3 4 5 (Strongly agree)

**4. Overall the instructor was an effective educator.**

(Strongly disagree) 1 2 3 4 5 (Strongly agree)

**5. The instructor explained difficult concepts clearly.**

(Strongly disagree) 1 2 3 4 5 (Strongly agree)

**6. What were the STRENGTHS of this simulation experience?**

**7. What were the WEAKNESSES of this simulation experience?**

**Thank you!**
