# Supplementary material for: In-Flight Emergency: A Simulation Case for Emergency Medicine Residents
Source: MedEdPORTAL. 2020 Aug 20;16:10949. doi: 10.15766/mep_2374-8265.10949 (PMC7449573; doi:10.15766/mep_2374-8265.10949)
Supplement: Supplementary file 1 — Simulation Case.docxSimulation Images.docxMedical Kit Supply List.docxCritical Actions Checklist.docxResident Evaluation.docxLearning Points.docx [file mep_2374-8265.10949-s001.zip › F. Learning Points.docx]

In-Flight Medical Emergencies

Medical emergencies occur in 1 of every 604 flights

On-board resources:

- First-aid kit
- Emergency medical kit with airway supplies, IV access supplies, medications
  - Airway supplies typically include BVM and OPAs
  - Though intubation equipment was on board for this case, this would be extremely unlikely on most commercial flights
- AED
- Flight attendants with training every other year in CPR and AEDs
- Ground-based medical consultation services
- Usually no obstetric/pediatric supplies

Do you have a legal obligation to step forward? Not in US, but ethical obligation as physician. However, on EU and Australian airlines physicians are legally obligated to respond.

Are you protected? Aviation Medical Assistance Act (AMAA) passed by Congress in 1998 offers legal protection to providers who respond to a medical emergency on American airliners.

“An individual shall not be liable for damages in any action brought in a Federal or State court arising out of the acts or omissions of the individual in providing or attempting to provide assistance in the case of an in-flight medical emergency *unless the individual, while rendering such assistance, is guilty of gross negligence or willful misconduct*.”

Can you accept gifts? Many airlines will offer an upgrade or trip voucher. However, AMAA does not comment on gifts, so Good Samaritan coverage may not apply if you do accept “payment.” Consider carefully.

5 C’s for Good Samaritan response:

- Competence- any physical or knowledge limitations? State medical qualifications to flight crew and patient
- Consent- from patient to assess and treat
- Communication- with patient, flight attendants, pilot, ground crew. Recommend diversion if patient’s condition is critical
- Collaborate- obtain medical equipment from flight attendant, ask other passengers for medications
- Clinical records- document encounter (treatment, vitals, etc.)

Flight diversion:

- 7% of medical emergencies require diversion (most commonly cardiac, respiratory, neurologic emergencies)
- Decision to divert lies solely with captain of aircraft (must consider fuel, location, medical resources)
- Costs anywhere from $15,000 to $600,000 depending on disruption

Flight environment:

- Cabin altitude of 5000-8000 ft
- Lower atmospheric pressure (gases expand): earache, sinus pain, abdominal pain, pneumothoraxes expand
- Lower partial pressure of gases (oxygen): worsening of pre-existing hypoxia. Passengers typically have a partial pressure of arterial oxygen of 40-60 mmHg (partial pressure of oxygen is normally 75-100 mmHg at sea level). Typical oxygen saturations are 88-93% at cruising altitudes.
- Lower humidity: reactive airway disease, mucosal dryness, dehydration

Medical emergencies:

Cardiac arrest:

- 0.3% of emergencies, 86% of deaths
- Compression-only CPR, use of AED
- Cessation of resuscitation efforts and pronouncement of death if ROSC not achieved within 20-30 minutes

Acute coronary syndromes:

- 8% of emergencies
- Aspirin, nitroglycerin, supplemental O2
- Consider immediate diversion

Stroke:

- 2% of emergencies
- Check blood glucose if possible (standard in-flight emergency medical kits do not contain a glucometer)

Altered mental status:

- Seizures: 5.8% of emergencies

Syncope:

- 37.4% of emergencies
- Altered eating patterns and fatigue from delayed flights may also contribute
- Measure blood pressure and pulse
- Laying the patient on the floor with the feet elevated may provide rapid relief

Dyspnea:

- 12% of emergencies
- Provide supplemental oxygen
- Albuterol MDI is available in medical kit
- In patients with COPD, the volume of gas in a non-communicating bulla will increase by 30% on ascent from sea level to 8000 ft
- A descent in altitude may permit higher pressures of oxygen, though at a risk of the use of more fuel, because fuel consumption is greater at lower altitudes
- Suspicion of pneumothorax in a patient who is in unstable condition may require the clinician to perform a needle thoracostomy, using the on-board equipment or even improvised (i.e. nonmedical) equipment if the patient is in an extremely unstable condition and the appropriate medical equipment is not available. Descending to a lower altitude may also be beneficial, because cabin pressure is inversely proportional to the altitude of the aircraft.

Extra tips/suggestions:

- Blood pressure measurement: use pulse
- Ask other passengers for medications and supplies (i.e. steroids, glucometer)
- Standard in-flight emergency kit does not contain a glucometer, though some airlines carry one as part of an enhanced emergency medical kit. Ask other passengers whether one of them has a glucometer. However, verification of calibration may not be possible. Cleanliness of the device and potential for transmission of bloodborne pathogens must also be considered.
- Can use clothes hanger as IV pole
- Move patient to first-class, aisle seat, or first row for more space if severely ill
- Intubate in prone or “straddle” position or fully reclined seat if in first class
- While you are providing medical assistance and may make recommendations, flight crew remains in ultimate command

References

Ho SF, Thirumoorthy T, Ng BB. What to do during inflight medical emergencies? Practice pointers from a medical ethicist and an aviation medicine specialist. Singapore Med J. 2017;58(1):14-17.

Wong, M. (2017). Doctor in the sky: Medico-legal issues during in-flight emergencies. Medical Law International, 17(1–2), 65–98. https://doi.org/10.1177/0968533217705693

DeLaney, M. “Is there a doctor on-board” 5 tips for dealing with in-flight emergencies. 2014. https://www.aliem.com/2014/01/doctor-on-board-5-tips-dealing-in-flight-emergencies/
