# Supplementary material for: In-Flight Emergency: A Simulation Case for Emergency Medicine Residents
Source: MedEdPORTAL. 2020 Aug 20;16:10949. doi: 10.15766/mep_2374-8265.10949 (PMC7449573; doi:10.15766/mep_2374-8265.10949)
Supplement: Supplementary file 1 — Simulation Case.docxSimulation Images.docxMedical Kit Supply List.docxCritical Actions Checklist.docxResident Evaluation.docxLearning Points.docx [file mep_2374-8265.10949-s001.zip › _Educational_Summary_Report_10949.pdf]

MedEdPORTAL® The AAMC Journal of Teaching and Learning Resources

Original Publication Open Access

In-Flight Emergency: A Simulation Case for Emergency Medicine Residents

Claire Hailey, MD*, Charles Lei, MD, Laurie Lawrence, MD

*Corresponding author: claire.hailey@vumc.org

Abstract

Introduction: In-flight medical emergencies are common occurrences that require medical professionals to manage patients in an unfamiliar setting with limited resources. Emergency medicine (EM) residents should be well prepared to care for patients in unusual environments such as on an aircraft. **Methods:** We developed a simulation case for EM residents featuring a 55-year-old male passenger who suffers a cardiac arrest secondary to a tension pneumothorax. We conducted this case eight times during a 5-hour block of scheduled simulation time. Participants included EM residents of all training levels from one residency program. We arranged the simulation lab as an airplane cabin, with rows of chairs representing airplane seats and a mannequin in a window seat as the patient. Residents were expected to manage cardiac arrest and perform needle thoracostomy on the patient. Residents also evaluated and treated a flight attendant with a near syncopal episode. Throughout the case, residents were expected to practice teamwork skills, including leadership, communication, situational awareness, and resource utilization. Participants were debriefed and completed voluntary anonymous evaluations of the session. **Results:** Seventeen EM residents participated in the simulation. Overall, all 17 found the simulation to be a valuable educational experience. In addition, all agreed or strongly agreed that they felt more prepared to respond to an in-flight emergency after participating in the simulation. **Discussion:** This simulation was determined to be a valuable part of EM resident education. The challenges presented and skills practiced in this in-flight medical emergency simulation case are transferable to other resource-limited environments.

Keywords
Emergency, Flight, Tension Pneumothorax, Pulseless Electrical Activity, Emergency Medicine, Airplane, In-Flight, Clinical/Procedural Skills Training, Simulation

Educational Objectives

By the end of this activity, learners will be able to:

1. Identify the resources available during an in-flight emergency.
2. Describe the role of a medical professional responding to an in-flight emergency.
3. Recognize the presentation of tension pneumothorax and describe its management.
4. Demonstrate the appropriate management of pulseless electrical activity cardiac arrest.
5. Demonstrate the appropriate initial evaluation and treatment of a patient with a near syncopal episode.

Citation:
Hailey C, Lei C, Lawrence L. In-flight emergency: a simulation case for emergency medicine residents. *MedEdPORTAL*. 2020;16:10949.
https://doi.org/10.15766/mep_2374-8265.10949

Introduction

A medical emergency occurs on an estimated one in every 604 flights.[1] In 2018, the United States Federal Aviation Administration's Air Traffic Organization handled over 16 million flights, suggesting that, on average, over 70 in-flight emergencies occurred in the US each day.[2] The frequency of these emergencies will continue to rise as commercial flight volumes increase. These medical emergencies are complicated by the altered physiology that takes place within a pressurized cabin and low partial pressure of oxygen.[1] In-flight emergencies are particularly difficult to manage because of the confined physical space and the limited access to medical supplies, medications, and trained personnel.

A multicenter survey of fourth-year medical students found that on average they felt unprepared to respond to in-flight emergencies.[3] Unfortunately, there are no standard curricula to bridge this known knowledge gap. Therefore, the majority of residents likely feel a similar unease when responding to an in-flight emergency. The use of simulation to recreate a medical emergency on an airplane has proven effective in teaching

Copyright © 2020 Hailey et al. This is an open-access publication distributed under the terms of the Creative Commons Attribution license.

the medical knowledge required to respond to an in-flight emergency.[4] Additionally, Pettit and Ferguson have published a case in *MedEdPORTAL* demonstrating the use of a simulated in-flight emergency as a team-building exercise.[5] Successfully managing a complex in-flight emergency requires both adequate medical knowledge and effective teamwork skills. Our simulation case adds to the available literature by combining elements of medical expertise and technical skill with core concepts in teamwork and communication in the management of an in-flight emergency.

Emergency medicine (EM) residents should be prepared to respond to an in-flight medical emergency. Training for an in-flight emergency also prepares EM residents for managing critically ill patients in a low-resource environment, an essential skill for graduating EM residents who may practice in limited-resource settings. This simulation case is designed specifically for EM residents. The most common medical emergencies encountered in-flight include syncope and respiratory complaints. This simulation case involves a patient with a cardiopulmonary emergency of tension pneumothorax and pulseless electrical activity (PEA) cardiac arrest. The patient has chronic obstructive pulmonary disease as a preexisting condition, which is acutely exacerbated by the altitude of the flight. Prior to this simulation case, participating residents should feel comfortable running an advanced cardiac life support resuscitation in the hospital environment. This simulation case requires residents to extend this knowledge to the resource-limited setting of an airplane.

Methods

Development

We developed this simulation case (Appendix A) for EM residents as a part of the simulation curriculum at Vanderbilt University Medical Center. We implemented the simulation in a 5-hour time block during our monthly EM residency simulation conference, and residents completed the case in small groups of two to three learners. Groups were composed of a mix of EM residents at different levels of training, from PGY 1 to PGY 3. We allotted 15 minutes for the case and 15 minutes for the debriefing, which occurred immediately after the simulation experience. We conducted the case a total of eight times. At the beginning of each academic year and prior to this simulation session, we provided residents with prerequisite knowledge of how to interact with the simulation personnel and mannequins as well as what to expect in the simulation lab.

Equipment/Environment

The simulation session took place at the Center for Experiential Learning and Assessment at Vanderbilt University School of Medicine. We placed rows of chairs on each side of a center aisle to simulate the cabin of an airplane, and positioned divider walls on either side of the chairs to simulate walls. We utilized a Laerdal SimMan 3G mannequin as the patient, although any high-fidelity adult mannequin could be used. We placed the mannequin in a window seat, dressed him in regular clothing, and placed a pack of cigarettes and an albuterol inhaler in his shirt pocket (Appendix B). Other supplies used in this case included an automated external defibrillator (AED) and an in-flight emergency medical kit containing specific medical supplies (Appendix C).

Personnel

Flight attendant 1 (actor): To start the case, flight attendant 1 announced, “Is there a doctor on board?” He/she assisted the medical team with tasks such as locating medical supplies, moving the ill passenger (e.g., to an aisle seat rather than window seat), and communicating with the pilot. Flight attendant 1 had basic first-aid training, including AED use, and assisted with performing chest compressions if requested.

Flight attendant 2 (actor): Flight attendant 2 had similar skills as flight attendant 1. Immediately after the medical team performed needle thoracostomy on the ill passenger, flight attendant 2 became very light-headed and developed palpitations, shortness of breath, and nausea. He/she had no recent symptoms of illness and no significant past medical history. Due to an inadequate oral intake of fluids, the stress of the in-flight emergency, and the viewing of the needle thoracostomy, flight attendant 2 had a vasovagal near syncopal episode. He/she stumbled and fell to the floor, without losing consciousness. The medical team had to also evaluate and treat him/her.

Pilot (voiceover by instructor or simulation technician): The pilot communicated and coordinated with the onboard medical team. He/she periodically requested an update on the status of the ill passenger. The pilot asked for an explanation when the medical team advised diverting the plane to the closest airport near a hospital. He/she informed the medical team that the aircraft was only 2 hours away from its destination and asked if the team could manage the passenger until they arrived at the destination. Given an adequate explanation, the pilot agreed with the recommendations of the medical team.

Ground-based medical service (voiceover by instructor or simulation technician): The ground-based medical service person communicated and coordinated with the onboard medical team. He/she asked for an explanation when the medical team advised diverting the plane to the closest airport near a hospital. He/she informed the medical team that the aircraft was only 2 hours

Copyright © 2020 Hailey et al. This is an open-access publication distributed under the terms of the Creative Commons Attribution license.

away from its destination and asked if the team could manage the passenger until they arrived at the destination. Given an adequate explanation, the ground-based physician agreed with the recommendations of the medical team.

Implementation
Before the start of the case, the EM residents were instructed to sit outside the simulation room in a row of chairs staged as the rear of an airplane. The case then started with a flight attendant making an announcement requesting medical assistance at the front of the airplane. Learners were expected to introduce themselves to the patient and flight attendants. They were able to obtain limited history from the patient due to his dyspnea. Participants were expected to request the emergency medical kit and AED from the flight attendant. They had to obtain vital signs manually (e.g., by feeling for a pulse and measuring a manual blood pressure) and perform a physical exam. The patient was hypotensive and tachycardic and had decreased left-sided lung sounds on auscultation, suggesting a tension pneumothorax. Residents were expected to obtain IV access and administer supplemental oxygen and bronchodilators. The patient then became unresponsive and pulseless. Residents were expected to initiate cardiopulmonary resuscitation and utilize the AED. Return of spontaneous circulation was obtained when the residents performed a needle thoracostomy to relieve the patient's tension pneumothorax. At that point, one of the flight attendants experienced a near syncopal episode. Learners were expected to measure the flight attendant's blood glucose or empirically administer glucose and IV or oral fluids. Throughout the case, residents were expected to communicate with the flight attendants, pilot, and ground-based medical staff to provide reasoning for diverting the aircraft. The case concluded with the airplane diverting to the nearest airport. The residents then participated in a debriefing of the case with simulation faculty.

Assessment
EM residents were assessed using a critical actions checklist that was developed through faculty consensus and based on the learning objectives for the case (Appendix D). Assessment was performed by a simulation faculty member who observed from the simulation control room. Debriefing of the case was performed immediately afterward by two simulation faculty members to provide the residents with immediate feedback based on the critical actions checklist. Residents were also given additional information on the medical management of the case during the debriefing session. Residents then completed anonymous paper evaluations of the simulation experience to provide an assessment of the simulation case and the faculty members' debriefing (Appendix E). This evaluation instrument was a standard form that had been approved by the Vanderbilt Institutional Review Board and has been used by our simulation faculty after each simulation session to obtain feedback from participants.

Debriefing
The debriefing session for this simulation took place immediately following each case. Debriefing was guided by two EM simulation faculty members who observed the case from the simulation control room. Advocacy-inquiry questioning was used to facilitate the discussion. Key topics for discussion included an overview of in-flight medical emergencies, patient care in resource-limited settings, and communication with other team members, including the pilot and ground-based medical staff. After the debriefing, residents received a document with additional learning points from the case (Appendix F) via email.

Results

We conducted this case eight times with a total of 17 learners in groups of two to three learners. All learners were EM residents ranging from PGY 1 to PGY 3 in level of training. Residents completed voluntary, anonymous surveys in which they rated aspects of the simulation experience on a 5-point Likert scale and provided comments on observed strengths and weaknesses of the session. All 17 (100%) residents completed the survey. All 17 (100%) respondents agreed or strongly agreed that the simulation was a valuable educational experience. Sixteen of 17 (94%) agreed or strongly agreed that the simulation scenario was realistic. In addition, all (100%) residents agreed or strongly agreed that they felt more prepared to respond to an in-flight emergency after participating in the simulation.

Qualitative comments included the following:

- "Realistic in the sense of making us sort through available resources and navigate the physical challenges of being on a flight."—PGY 2 resident
- "Great reminder about common in-flight emergencies, favorite sim of the year."—PGY 3 resident
- "Really fun, made me feel more prepared for this happening."—PGY 2 resident

Other participants commented on the usefulness of the debriefing for discussing elements of teamwork and communication, as well as the specific role of ground-based medical staff. Residents especially appreciated the challenge of managing a critically ill patient in an unusual environment with limited resources. Participants found no weaknesses in this case.

Copyright © 2020 Hailey et al. This is an open-access publication distributed under the terms of the Creative Commons Attribution license.

Discussion

We developed and implemented a simulation case for EM residents featuring an in-flight medical emergency in which a passenger developed PEA arrest secondary to tension pneumothorax and a flight attendant had a near syncopal event. We found this case to be impactful because it required residents to manage patients in an unfamiliar environment with limited resources. The case gave residents an opportunity to practice key crisis resource-management skills, including team leadership, communication, and situational awareness. The need to simultaneously manage two patients required learners to utilize and prioritize all available resources. Residents also gained valuable experience coordinating with different types of personnel, including the flight attendants, pilot, and ground-based medical staff. All participants rated the simulation experience positively and felt more prepared to respond to an in-flight emergency.

One of the primary challenges encountered during implementation of this case was optimizing the realism of the scenario. For example, during an actual in-flight medical emergency, medical responders would have to manually measure a patient's vital signs, including blood pressure, heart rate, and respiratory rate. Thus, we looked for ways to provide participants with updated vital signs in a timely manner. We experimented with having the flight attendant periodically announce the patient's vital signs but noticed that this method significantly decreased the realism of the role of the flight attendant. Ultimately, we utilized a mobile application (SimMon, Castle Anderson ApS) to display vital sign updates on a tablet device. In order to receive these updates, participants had to clearly indicate that they were planning to reassess the patient's vital signs (e.g., by inflating the blood pressure cuff, palpating the carotid artery, or auscultating the chest). Although a vital sign monitor would normally not be available on an airplane, we found that use of the mobile application in this manner did not negatively impact the realism of our scenario.

As another example, medical responders during an actual in-flight emergency would have to manage a patient in the confined space of an airplane cabin. In an attempt to recreate these conditions in our simulation lab, we placed the rows of chairs close together and created a narrow aisle for participants to use. It was not possible to replicate the low ceilings of an airplane cabin. In the first several groups, we witnessed residents attempting to move or climb over the chairs to gain better access to the ill passenger. Thus, we taped the chairs to the floor and reminded participants during the case, through the flight attendant, that it would be physically impossible to rearrange the chairs or climb from one row to another in real life. To increase environmental realism, we posted images of aerial views out of the airplane windows on the divider walls. We also experimented with playing ambient airplane noises in the simulation room but quickly found that these sounds made it difficult for the simulation faculty to hear participants from the control room.

During implementation of this simulation scenario, we learned that residents had limited understanding of the resources available on a commercial flight, such as the contents of the emergency medical kit or the availability of an AED. In addition, many residents were uncertain of the obligations and limitations of their role as medical professionals during an in-flight emergency. Overall, residents most appreciated the opportunity to practice core crisis resource-management skills. We were able to review all of these topics in the debriefing sessions.

The primary limitation of this simulation case was that it presented a rare in-flight medical scenario. While in-flight emergencies are common, most do not require flight diversion. Near syncope, the most frequently encountered medical issue, can almost always be managed until the flight reaches its destination. In contrast, tension pneumothorax with PEA arrest is a rare but true in-flight emergency that requires immediate resuscitation and diversion to the nearest hospital. Despite its rarity, our simulation scenario remains generalizable because it trains EM residents to respond to all in-flight emergencies and helps them develop key crisis resource-management skills that will enable them to respond to any medical emergency in resource-limited settings. In addition, this simulation scenario is also generalizable to other learner groups. In-flight emergencies may be encountered by both non-EM physicians and other medical personnel, such as medical students and nurses. This case can be adapted and utilized by other learner populations to train resource-management skills.

Overall, participating EM residents found this case to be a valuable educational experience. We will continue to include this case in our simulation curriculum to train future EM residents at our institution.

Appendices

A. Simulation Case.docx

B. Simulation Images.docx

Copyright © 2020 Hailey et al. This is an open-access publication distributed under the terms of the Creative Commons Attribution license.

C. Medical Kit Supply List.docx

D. Critical Actions Checklist.docx

E. Resident Evaluation.docx

F. Learning Points.docx

All appendices are peer reviewed as integral parts of the Original Publication.

Claire Hailey, MD: Assistant Professor of Emergency Medicine, Department of Emergency Medicine, Vanderbilt University Medical Center

Charles Lei, MD: Assistant Professor of Emergency Medicine, Department of Emergency Medicine, Vanderbilt University Medical Center

Laurie Lawrence, MD: Associate Professor of Emergency Medicine and Pediatrics, Department of Emergency Medicine, Vanderbilt University Medical Center

Disclosures

None to report.

Funding/Support

None to report.

Ethical Approval

Reported as not applicable.

References

1. Martin-Gill C, Doyle TJ, Yealy DM. In-flight medical emergencies: a review. *JAMA*. 2018;320(24):2580-2590. https://doi.org/10.1001/jama.2018.19842
2. Air traffic by the numbers. Federal Aviation Administration. *Accessed December* 28, 2019. https://www.faa.gov/air_traffic/by_the_numbers/
3. Katzer RJ, Duong D, Weber M, Memmer A, Buchanan I. Management of in-flight medical emergencies: are senior medical students prepared to respond to this community need? *West J Emerg Med*. 2014;15(7):925-929. https://doi.org/10.5811/westjem.2014.9.22569
4. Padaki A, Redha W, Clark T, et al. Simulation training for in-flight medical emergencies improves provider knowledge and confidence. *Aerosp Med Hum Perform*. 2018;89(12):1076-1079. https://doi.org/10.3357/AMHP.4945.2018
5. Pettit J, Ferguson KJ. Medical emergency during flight: a team-building exercise. *MedEdPORTAL*. 2017;13:10530. https://doi.org/10.15766/mep_2374-8265.10530

Received: June 10, 2019
Accepted: January 30, 2020
Published: August 20, 2020

Copyright © 2020 Hailey et al. This is an open-access publication distributed under the terms of the Creative Commons Attribution license.
