# Supplementary figures and images for: In-Flight Emergency: A Simulation Case for Emergency Medicine Residents
Source: MedEdPORTAL. 2020 Aug 20;16:10949. doi: 10.15766/mep_2374-8265.10949 (PMC7449573; doi:10.15766/mep_2374-8265.10949)

**Appendix B**


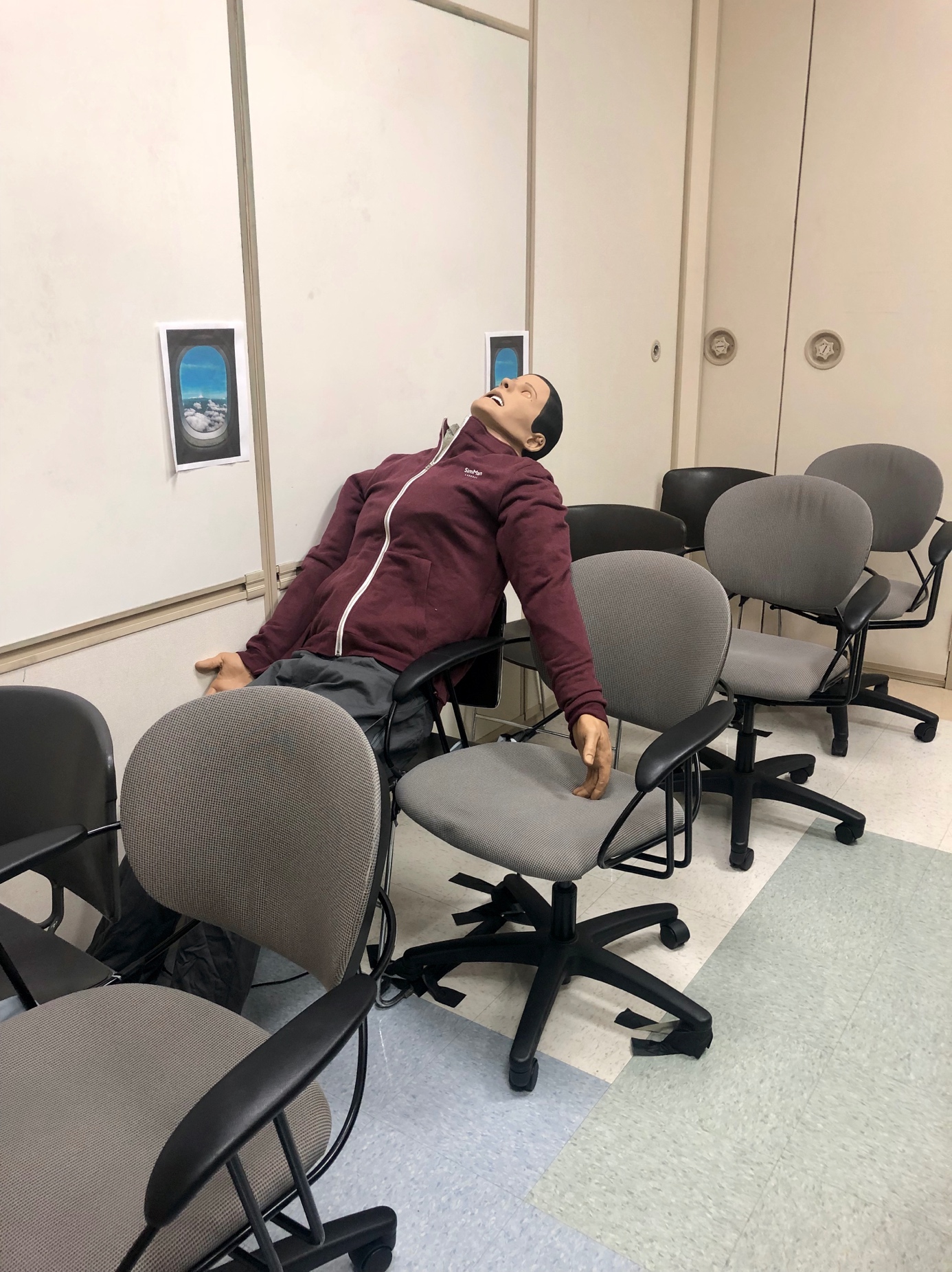


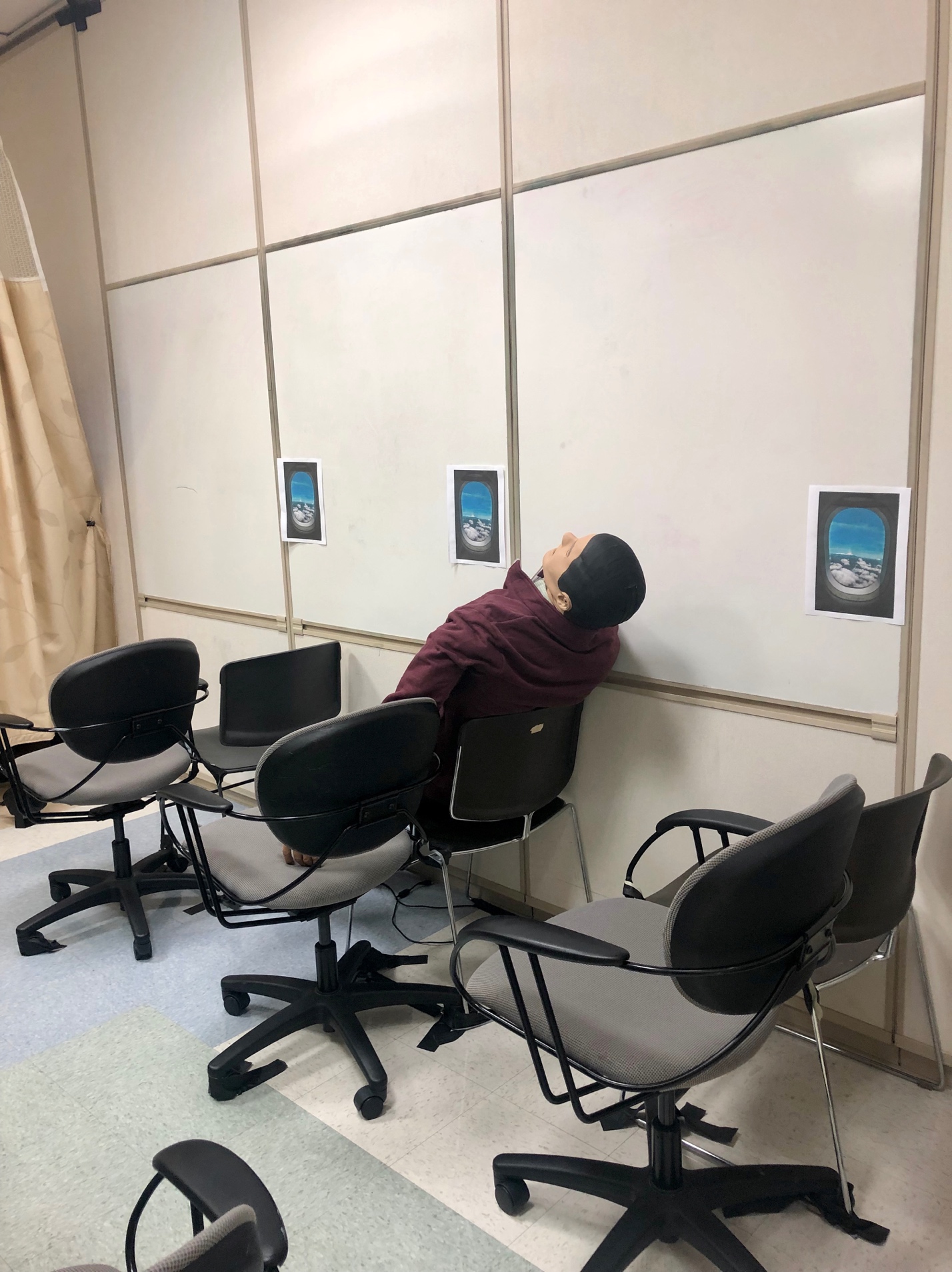

Supplement: Supplementary file 1 — Simulation Case.docxSimulation Images.docxMedical Kit Supply List.docxCritical Actions Checklist.docxResident Evaluation.docxLearning Points.docx [file mep_2374-8265.10949-s001.zip › B. Simulation Images.docx]
